# A Rare Case of Adult-Onset Bilateral Nonspecific Orbital Inflammation (NSOI) With Concomitant Unilateral Third Cranial Nerve Palsy

**DOI:** 10.7759/cureus.67181

**Published:** 2024-08-19

**Authors:** Haider Ghumman, Ghazaleh Baradaran-Rafii, Anosh Dadabhoy, Ussama Ghumman, Christine G Saad

**Affiliations:** 1 Ophthalmology, University of South Florida (USF) Health Morsani College of Medicine, Tampa, USA; 2 Ophthalmology, Irvine Valley College, Irvine, USA; 3 Transplant Hepatology, University of Texas Health Science Center at San Antonio, San Antonio, USA; 4 Ophthalmology, Lehigh Valley Health Network, Allentown, USA

**Keywords:** third cranial nerve (oculomotor nerve) palsy, orbital inflammatory syndrome, idiopathic orbital inflammatory disease, nonspecific orbital inflammation, orbital pseudotumor

## Abstract

Nonspecific orbital inflammation (NSOI), also known as orbital pseudotumor, is a condition characterized by inflammation in the tissues around the eye socket (orbit) without a clearly identifiable cause. This inflammatory disorder can affect various structures within the orbit, including muscles, fat, and connective tissues, leading to symptoms such as pain, swelling, and changes in vision. A 74-year-old man with a history of previous orbital trauma presented with acute-onset head and orbital pain, followed by restricted left eye movements in all directions, left ptosis, and a dilated left pupil. Orbital imaging revealed bilateral inflammation of the lateral rectus muscles and orbital fat, suggestive of bilateral NSOI, while brain and laboratory studies ruled out other differential diagnoses. The presence of left ptosis, a dilated pupil, and limited upward, downward, and inward movements in the left eye suggested intraorbital involvement of both the superior and inferior divisions of the left third nerve. The complete resolution of orbital symptoms and third nerve function after systemic corticosteroid therapy supported the inflammatory nature of the nerve involvement in this case. The case is notable in terms of bilateral involvement in adult-onset NSOI, the possible role of previous orbital trauma in the development of the disease, and the inflammatory involvement of third nerve divisions following the extension of inflammation into the orbital tissues. NSOI can mimic other, more serious conditions, making accurate diagnosis crucial for effective management and treatment. Understanding its presentation, potential causes, and appropriate diagnostic approaches is essential in providing optimal care for patients affected by this complex inflammatory condition.

## Introduction

Nonspecific orbital inflammation (NSOI), which includes terms such as orbital pseudotumor, idiopathic orbital inflammation (IOI), and orbital inflammatory syndrome (OIS), is a space-occupying condition marked by orbital inflammation with no identifiable infectious, systemic, or malignant origin [1]. NSOI presents with variable clinical manifestations, from localized forms targeting the extraocular muscles (orbital myositis), lacrimal gland (dacryoadenitis), and sclera (scleritis), to widespread and diffuse involvement of orbital fatty tissues [1].

The primary manifestations of NSOI include eyelid erythema and swelling, intense orbital pain, and noticeable proptosis [2]. Painful eye movements and diplopia are commonly present, indicating involvement of the extraocular muscles [3]. While optic nerve compression can occur in severe cases with visual impairment, inflammatory or compressive involvement of other cranial nerves is not considered a feature of NSOI [4].

In this context, we present a case of adult-onset bilateral NSOI with a distinctive clinical presentation of unilateral pupil-sparing third nerve palsy, alongside a history of previous orbital trauma. This case underscores the variability in NSOI's clinical expression and highlights the importance of a comprehensive evaluation to differentiate it from other orbital pathologies and guide appropriate management strategies. Understanding the diverse presentations of NSOI is crucial for accurate diagnosis and optimal patient care in clinical practice.

## Case presentation

A 74-year-old patient with a past medical history of chronic headache, multi-nodal nontoxic goiter, and gastroesophageal reflux disorder presented with an eight-day history of worsening left eye tearing and severe headache. The headache had a sudden onset, was intense in severity, intermittent, and radiated to the left eye. Within a few days of the headache, the patient developed left ptosis and diplopia, adding to his symptoms. He did not report any reduction in vision in either eye. The diplopia was binocular and present in all directions of gaze. There was no history of fever, neck rigidity, preceding infection, or any other systemic illness. The patient reported a previous moderate reduction in vision in the right eye, which he was informed was due to a retinal disorder. Additionally, he had a history of orbital trauma two and a half years prior to the current presentation, which led to symptoms and signs of transient pain behind the right eye and temporary abnormal physical dipping of the right eye. A magnetic resonance imaging (MRI) of the brain and a computed tomography (CT) scan were performed at that time, both of which showed normal results, and the symptoms improved over time without any permanent defects.

Upon ophthalmology consultation for his current presentation, the visual acuity in the right eye was 20/20 and 20/40 in the left eye, with pressures of 18 mmHg and 21 mmHg, respectively. The right eye had normal extraocular movements in all directions, except for a mild reduction in abduction. There were severe limitations in upward gaze, downward gaze, and adduction in the left eye, while abduction was slightly affected. Pupillary and visual field examinations were normal in both eyes. Upon slit lamp and fundus examination, the right eye displayed normal findings, except for a nuclear sclerosing cataract and an old lamellar hole in the fovea. However, there was complete ptosis in the left eye, in addition to a nuclear sclerosing cataract. There was no eyelid edema, prominent proptosis, or audible bruit over the eyes. The remainder of the neurological examination was normal.

**Figure 1:**
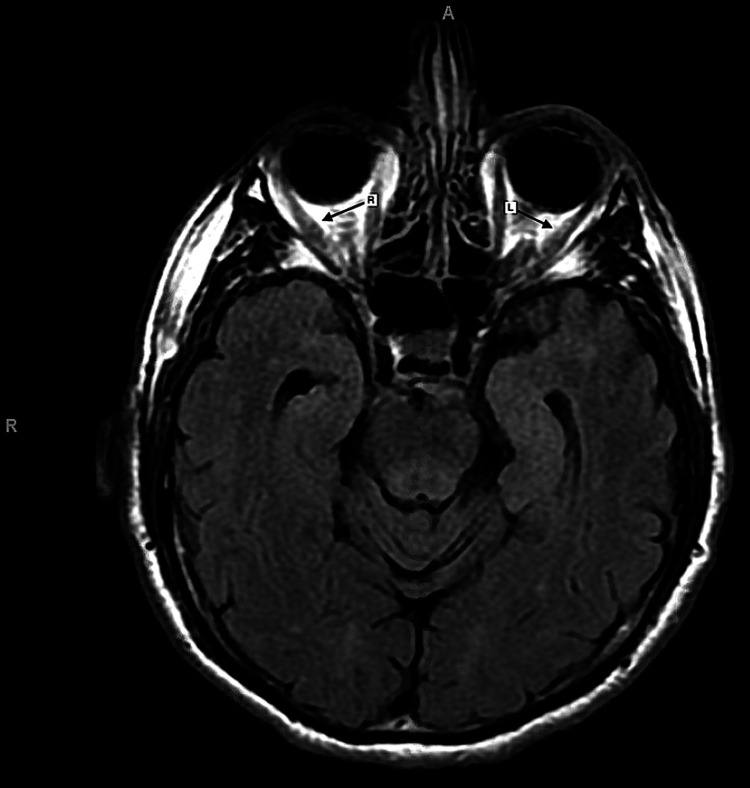
MRI of the orbits depicting lateral rectus muscle belly enhancement "L": Arrow to the left lateral rectus muscle; "R": Arrow to the right lateral rectus muscle

**Figure 2:**
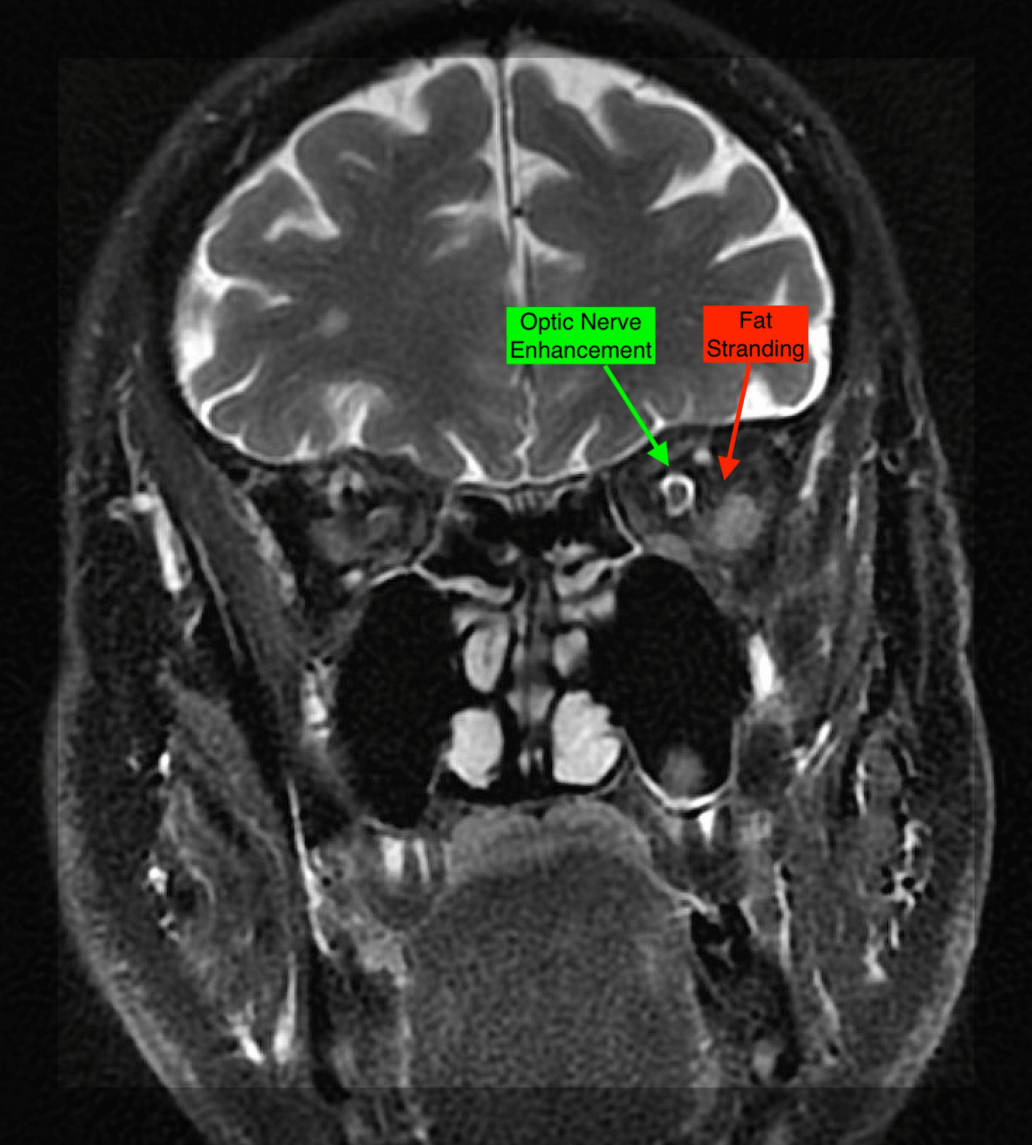
T2 fat-suppressed MRI image depicting left eye fat stranding and increased optic nerve sheath signal

Laboratory and imaging studies were done based on a list of differential diagnoses, including thyroid eye disease, orbital cellulitis, orbital inflammation, orbital neoplasm, neuromuscular disorders, intracranial third nerve palsy, and lymphoproliferative infiltration. Laboratory investigations, including complete blood count, blood glucose level, liver and renal tests, and thyroid function tests, all returned normal results. MRI of the brain was normal, while MRI of the orbits revealed asymmetric enhancement and swelling of the left lateral rectus muscle, with a lesser extent of swelling in the right lateral rectus muscle (Figure 1), increased fat stranding within the left intraconal orbital space more than the right (Figure 2), and slightly increased signal within the optic nerve sheaths of the left, more so than the right (Figure 2). These findings were highly suspicious for bilateral NSOI, with the left orbit being more affected than the right. The abnormal imaging findings did not extend to the orbital apex or cavernous sinuses.

The patient was started on intravenous steroids, followed by oral prednisolone and a slow tapering over six weeks. On a three-month follow-up, the patient’s symptoms significantly improved, with complete resolution of eye motility and ptosis.

## Discussion

NSOI is the third most common orbital disease, following thyroid eye disease and lymphoproliferative disorders [5]. It accounts for 8% to 11% of all orbital tumors and is more common in adults, especially middle-aged women [3,6]. NSOI is characterized by a diverse combination of cellular infiltrates, predominantly lymphocytes, plasma cells, and eosinophils, accompanied by variable levels of reactive fibrosis [1]. It presents with a wide range of clinical manifestations, spanning from localized (myositis, dacryocystitis, scleritis, uveitis, etc.) to diffuse forms (orbital fat inflammation). The imaging features are crucial for diagnosing NSOI and may reveal enlargement of extraocular muscles, inflammation of orbital fat, swelling of the lacrimal gland, and potential extension into the orbital apex and cavernous sinus [7]. Rapid resolution of symptoms following corticosteroid therapy is another characteristic of NSOI with diagnostic value. Systemic administration of prednisolone typically results in a dramatic response within 48 hours [8]. Although NSOI most commonly presents bilaterally in children and unilaterally in adults, this report presents a rare case of adult-onset bilateral NSOI diagnosed based on typical clinical presentation and imaging findings. The diagnosis was further supported by the absence of concurrent systemic disease, normal brain MRI results, and a favorable response to steroid therapy.

Immune-mediated, infectious, and traumatic etiologies have been implicated in the pathophysiology of NSOI. Different types of NSOI have been observed in association with various rheumatologic conditions, such as Crohn's disease, ankylosing spondylitis, rheumatoid arthritis, and myasthenia gravis, possibly due to a dysregulation in innate and adaptive immunity [9,10]. In terms of infections, the theory of molecular mimicry may be responsible for triggering orbital inflammation after an acute infection, such as an upper respiratory tract infection [11]. In our case, there was no history of immune-mediated disease or recent infection that could explain the onset of the disease. However, there was a history of previous orbital trauma. It has been proposed that trauma may lead to increased vascular permeability, causing the release of antigenic substances that initiate an inflammatory cascade [12]. While it is not possible to establish a definitive causal relationship, it may be hypothesized that the history of orbital trauma in our patient could have played a role in the onset of NSOI.

Interestingly, our patient presented with unilateral ptosis, double vision, and restricted left eye movement in all directions. A reason for limited ocular movement in NSOI is the restrictive involvement of the extraocular muscles [1]. In our patient, the involvement of the left lateral rectus explains the limited abduction of the left eye. However, the restricted upward, downward, and adduction movements cannot be explained by this, as imaging shows no involvement of other muscles. The clinical presentation of normal pupillary function, along with limited upward, downward, and inward movements of the left eye, strongly supports pupil-sparing third cranial nerve palsy. While orbital pseudotumor typically remains confined to the orbit, in rare instances it can extend to the middle cranial fossa and cavernous sinus, affecting multiple cranial nerves. MRI findings in our case did not show evidence of involvement in these areas, making the third nerve palsy anatomically unique and not easily explained. It is possible that diffuse inflammation extended to the muscle bellies of the associated muscles, causing deficits in movement. Another possibility is that diffuse inflammation affected the terminal portion of the oculomotor nerve, leading to the observed movement abnormalities. Orbital apex diseases require a broad differential, as done in this case, due to the variety of possible etiologies. Possible etiologies can be inflammatory (sarcoidosis, systemic lupus erythematosus, Churg-Strauss syndrome, etc.), infectious (fungal, bacterial, spirochetes, viruses), neoplastic, iatrogenic/traumatic, vascular (carotid-cavernous aneurysm, carotid-cavernous fistula, cavernous sinus thrombosis), mucocele, fibrous dysplasia, and neurofibromatosis [13]. The most common initial presentation for orbital apex syndromes is visual loss and ophthalmoplegia involving multiple cranial nerves [13]. Appropriate investigation of the potential differential diagnoses is necessary to ensure that appropriate management is pursued.

The oculomotor nerve exits the cranial cavity via the superior orbital fissure, dividing into superior and inferior branches. The superior branch innervates the superior rectus and levator palpebrae superioris, accompanied by sympathetic fibers for the superior tarsal muscle. The inferior branch supplies motor innervation to the inferior rectus, medial rectus, and inferior oblique muscles, and provides parasympathetic fibers to the ciliary ganglion, controlling the sphincter pupillae and ciliary muscles [14]. A previous report described a case of unilateral NSOI where the superior division of the third nerve was affected by orbital inflammation, causing ptosis and limited upward eye movement in a young woman [15]. Our case may represent the second report of third nerve involvement in NSOI, but notably, the involvement of both superior and inferior divisions distinguishes our case from the previous report. The presence of limited upward eye movement and complete ptosis suggests the involvement of the superior division, while restricted inward and downward movement, with dilated pupils, suggests the involvement of the inferior division. Concurrent involvement of both superior and inferior divisions favors diffuse orbital inflammation.

## Conclusions

This case underscores the diagnostic challenge posed by NSOI, particularly in its less common presentations, such as unilateral pupil-sparing third nerve palsy. The variability in clinical manifestations of NSOI, ranging from localized forms affecting specific orbital structures to diffuse inflammation involving orbital fat, highlights the necessity of a comprehensive evaluation to distinguish NSOI from other orbital pathologies. Prompt recognition is crucial, as NSOI typically responds dramatically to systemic corticosteroid therapy, emphasizing the importance of early intervention to mitigate potential complications and ensure optimal patient outcomes. Further research and case reports are warranted to enhance our understanding of NSOI's diverse presentations and refine management strategies tailored to individual patient needs. This case serves as a reminder to consider NSOI in the differential diagnosis of acute orbital syndromes presenting with unusual neurological deficits, enabling timely and effective treatment interventions.
